# Medication adherence and cognitive performance in schizophrenia-spectrum and bipolar disorder: results from the PsyCourse Study

**DOI:** 10.1038/s41398-023-02373-x

**Published:** 2023-03-25

**Authors:** Fanny Senner, Lena Hiendl, Susanne Bengesser, Kristina Adorjan, Ion-George Anghelescu, Bernhardt T. Baune, Monika Budde, Udo Dannlowski, Detlef E. Dietrich, Peter Falkai, Andreas J. Fallgatter, Alkomiet Hasan, Maria Heilbronner, Markus Jäger, Georg Juckel, Janos L. Kalman, Carsten Konrad, Mojtaba Oraki Kohshour, Sergi Papiol, Daniela Reich-Erkelenz, Jens Reimer, Sabrina K. Schaupp, Max Schmauß, Simon Senner, Carsten Spitzer, Thomas Vogl, Jörg Zimmermann, Urs Heilbronner, Eva C. Schulte, Thomas G. Schulze, Eva Z. Reininghaus, Sophie-Kathrin Kirchner, Nina Dalkner

**Affiliations:** 1 Department of Psychiatry and Psychotherapy, University Hospital, LMU Munich, Munich, 80336 Germany; 2 Institute of Psychiatric Phenomics and Genomics, University Hospital, LMU Munich, Munich, 80336 Germany; 3 Department of Psychiatry and Psychotherapeutic Medicine, Research Unit for Neurobiology and Anthropometrics in Bipolar Affective Disorder, Medical University of Graz, Graz, 8036 Austria; 4 Department of Psychiatry and Psychotherapy, Mental Health Institute Berlin, Berlin, 14050 Germany; 5 Department of Psychiatry, University of Münster, Münster, 48149 Germany; 6 Department of Psychiatry, Melbourne Medical School, The University of Melbourne, Melbourne, VIC Australia; 7 Institute for Translational Psychiatry, University of Münster, Münster, 48149 Germany; 8 AMEOS Clinical Center Hildesheim, Hildesheim, 31135 Germany; 9 Center for Systems Neuroscience (ZSN), Hannover, 30559 Germany; 10 Department of Psychiatry, Medical School of Hannover, Hannover, 30625 Germany; 11 Department of Psychiatry and Psychotherapy, Tübingen Center for Mental Health, University of Tübingen, Tübingen, 72076 Germany; 12 Department of Psychiatry, Psychotherapy and Psychosomatics, Faculty of Medicine, University of Augsburg, Bezirkskrankenhaus Augsburg, Augsburg, 86156 Germany; 13 Department of Psychiatry II, Ulm University, Bezirkskrankenhaus Günzburg, Günzburg, 89312 Germany; 14 Department of Psychiatry, Ruhr University Bochum, LWL University Hospital, Bochum, 44791 Germany; 15 Department of Psychiatry and Psychotherapy, Agaplesion Diakonieklinikum, Rotenburg, 27356 Germany; 16 Department of Immunology, Faculty of Medicine, Ahvaz Jundishapur University of Medical Sciences, Ahvaz, Iran; 17 Department of Psychiatry and Psychotherapy, University Medical Center Hamburg-Eppendorf, Hamburg, 20246 Germany; 18 Department of Psychiatry, Health North Hospital Group, Bremen, 28102 Germany; 19 Center for Psychiatry Reichenau, Academic Hospital University of Konstanz, Konstanz, 78479 Germany; 20 Department of Psychosomatic Medicine and Psychotherapy, University Medical Center Rostock, Rostock, 18147 Germany; 21 Psychiatrieverbund Oldenburger Land gGmbH, Karl-Jaspers-Klinik, Bad Zwischenahn, 26160 Germany; 22 Department of Psychiatry and Psychotherapy, University Hospital Bonn, Medical Faculty, University of Bonn, Bonn, 53105 Germany; 23 Institute of Human Genetics, University Hospital Bonn, Medical Faculty, University of Bonn, Bonn, 53127 Germany; 24 Department of Psychiatry and Behavorial Sciences, SUNY Upstate Medical University, Syracuse, 54 NY USA

**Keywords:** Schizophrenia, Bipolar disorder

## Abstract

Existing guidelines recommend psychopharmacological treatment for the management of schizophrenia and bipolar disorder as part of holistic treatment concepts. About half of the patients do not take their medication regularly, although treatment adherence can prevent exacerbations and re-hospitalizations. To date, the relationship between medication adherence and cognitive performance is understudied. Therefore, this study investigated the relationship between medication adherence and cognitive performance by analyzing the data of 862 participants with schizophrenia-spectrum and bipolar disorders (mean [SD] age, 41.9 [12.48] years; 44.8% female) from a multicenter study (PsyCourse Study). Z-scores for three cognitive domains were calculated, global functioning was measured with the Global Assessment of Functioning Scale, and adherence was assessed by a self-rating questionnaire. We evaluated four multiple linear regression models and built three clusters with hierarchical cluster analyses. Higher adherence behavior (*p* < 0.001) was associated with better global functioning but showed no impact on the cognitive domains *learning and memory*, *executive function*, and *psychomotor speed*. The hierarchical cluster analysis resulted in three clusters with different cognitive performances, but patients in all clusters showed similar adherence behavior. The study identified cognitive subgroups independent of diagnoses, but no differences were found in the adherence behavior of the patients in these new clusters. In summary, medication adherence was associated with global but not cognitive functioning in patients with schizophrenia-spectrum and bipolar disorders. In both diagnostic groups, cognitive function might be influenced by various factors but not medication adherence.

## Introduction

Psychiatric disorders account for 7% of the overall global burden of diseases, as measured in disability-adjusted life years [1]. Globally, schizophrenia is the most common psychotic disorder and has a prevalence of 0.6–1% [2]. Bipolar disorder has a prevalence of 3–5% [2, 3]. The two disorders show a high overlap in terms of symptoms and genetic bases [4]. Cognitive impairment is frequent in both disorders and contributes to reduced social and occupational functioning; however, the patterns, degrees, and frequencies of cognitive deficits differ between schizophrenia and bipolar disorder [2, 5].

In schizophrenia, cognitive impairments are one of the core features and a major contributor to lower social and occupational functioning [2, 5]. Cognitive deficits affect up to 70% of patients with schizophrenia [6], and almost every aspect of cognition (e.g., attention, memory, and language) is impaired, although individual impairment varies. These deficits usually appear in late childhood or early adolescence and often before the actual onset of schizophrenia [7] and thus before treatment with antipsychotic drugs [8].

In bipolar disorder, 40–60% of patients suffer from cognitive disturbances [9]. Neurocognitive endophenotypes are found in unaffected relatives of people with schizophrenia, but the data on patients with bipolar disorder are less clear: One study did not detect neurocognitive endophenotypes in unaffected relatives, but a meta-analysis suggested that first-degree relatives of patients with bipolar disorder demonstrate poorer cognitive functioning than healthy controls [10, 11]. During the course of a bipolar disorder, subtle but substantial neurocognitive deficits can be found across all mood states [12]. These deficits occur with high effect sizes, but are less pronounced compared to those in schizophrenia [13]. Most of these deficits seem to remit during periods of euthymia, but some of them may persist in approximately one third of bipolar patients [14]. They seem to be related to disease severity, the presence of psychotic symptoms, prolonged duration of illness, more manic episodes, and subsyndromal depressive symptoms [15].

Schizophrenia-related cognitive deficits are considered to be robust [5, 16], whereas cognitive deficits in patients with bipolar disorder seem to be more state related [17]. Children at risk of developing schizophrenia have lower cognitive performance levels than controls by the time they enter school, and the gap increases over time [18]. On the contrary, the school performance of children with a high risk of developing bipolar disorder even exceeds that of their peers [18]. Individuals with schizophrenia and bipolar disorder appear to experience further declines in cognitive function after onset of the disease, but the extent of the impairments is greater in schizophrenia than in bipolar disorder [19].

In both disorders, psychopharmacological treatment is the number one treatment recommendation across all guidelines [2, 3]; however, psychopharmacological treatment appears to have only limited effects on cognitive performance [2, 3, 5]. Within the last fifty years, effective medication has been discovered: it is capable of reducing several symptom domains in both schizophrenia-spectrum and bipolar disorders, but it has no established direct effects on cognition. Aggravating is the fact that a substantial number of patients does not take their medication regularly [20, 21], reducing the likelihood for long-standing remission and recovery. Nonadherence ranges from 44 to 56% in patients from the affective to psychotic spectrum [21]. The World Health Organization defines medication nonadherence as “a case in which a person’s behavior in taking medication does not correspond with agreed recommendations from health personnel” [22]. The degree of medication adherence is therefore an important indicator of whether the patient is medicated or not. Nonadherence can be measured by various means, including self-rating instruments, external assessment interviews, or therapeutic drug monitoring of plasma levels. Questionnaires like the Medication Adherence Report Scale (MARS) are effective as an self-report tool for measuring patients’ reports of their medication [23]. Questionnaires like the Clinician Rating Scale (CRS) and the Brief Adherence Rating Scale (BARS) check the regularity of medication intake by professional assessment [24, 25]. The measurement of the regularity of medication intake makes it possible to have a graduated view at the patients’ handling of the prescribed medication. In our study, we evaluated medication adherence with a self-rating questionnaire with assemblance to BARS. Nonadherence can have various reasons and be intentional or unintentional. The consequences of nonadherence include relapses, recurrences, suicidal tendencies, frequent hospitalizations, and an unfavorable course of disease with a reduced quality of life [21]. In addition, nonadherence increases the socioeconomic burden of the disease and leads to imbalances in the use of precious resources [26]. Various factors pose a risk of nonadherence, and an essential role is played by socio-demographic characteristics, such as unemployment, educational attainment, and age; comorbidities and substance abuse; treatment-related factors, such as adverse effects and drug treatment complexity; and the quality of the doctor-patient relationship [21]. Further important factors, especially among psychiatric patients, are attitudes towards drugs, perceived stigma, and lack of understanding of the disease and the cognitive impairment [21, 27].

The evidence for a relationship between cognitive deficits and treatment adherence remains sparse. Eight of 18 studies comparing adherence behavior and cognition in patients with schizophrenia revealed a positive correlation between cognitive performance and medication adherence, but the other ten studies did not [28]. The heterogeneous results may be explained by the small sample sizes of the selected studies (*n*_max_ = 184 participants) and the different methods used to measure adherence.

The present study aimed to clarify the relationship between medication adherence and cognitive function by analyzing data from the PsyCourse Study, a large, multi-center, transdiagnostic sample of deeply phenotyped patients with schizophrenia-spectrum and affective disorders who underwent thorough cognitive testing [29]. To do so, the study focused on the following research questions: (1) Does medication adherence influence cognitive and global functioning in patients with schizophrenia-spectrum and bipolar disorders? (2) Does the association between medication adherence and cognitive function depend on the diagnostic group? (3) Do specific cognitive clusters exist independent of diagnoses, and if so, and are there differences in the adherence behavior of patients in these clusters? We hypothesized that higher medication adherence is associated with a higher level of cognitive and global function in both diagnostic groups and that there are specific cognitive clusters that differ in adherence behavior.

## Patients and methods

### Study sample

This study used data from the first study visit (date of release: March 2020, version 4.0) from the longitudinal, naturalistic, multi-center PsyCourse Study, which was conducted in Germany and Austria (www.PsyCourse.de) between 2011 and 2019 [29]. This project aims on identifying clinical, neurobiological, and molecular genetic signatures of the longitudinal course of major psychiatric disorders. A vast battery of clinical and biological data for many potential research questions has been collected. Diagnoses were made with parts of the Structured Clinical Interview for DSM-IV (SCID) [30]. Eligible participants (*n* = 862; 44.8% female, 55.2% male) were individuals with a schizophrenia-spectrum disorder (schizophrenia, other psychotic disorder, schizoaffective disorder; *n* = 439) or bipolar disorder (*n* = 423) with existing information on medication adherence behavior and neurocognitive testing results. Comprehensive phenotypic data, such as sociodemographics, illness history, neurocognitive performance, psychopathology, and functioning, were assessed. A detailed description of the study design is available in the publication by Budde et al. [29]. All participants gave written informed consent. The study was approved by the responsible ethics committee and confirmed with the Declaration of Helsinki.

### Neurocognitive assessment

Neurocognitive testing was performed by trained raters. The domains *learning and memory*, *executive function*, and *psychomotor speed* were assessed with the following tests: Trail Making Test (TMT) [31], Verbal Digit Span (VDS) [32], and Digit Symbol Test (DST) [33]. The multiple-choice vocabulary intelligence test (MWT-B) was used to assess verbal intelligence, an approximate measure of general intelligence [34]. Detailed description is available in the Supplementary Material 1.

### Measurement of adherence

Adherence was measured with a self-assessment questionnaire that asked whether the patient had taken their psychopharmacological medication as prescribed in the last seven days and the last six months [27]. The questionnaire was self-constructed and non-standardized. The response options were as follows: 1, every day, exactly as prescribed; 2, every day, but not always as prescribed; 3, regularly, but not every day; 4, sometimes, but not regularly; 5, rarely; and 6, not at all. For the present study, we used only the information on adherence behavior in the last six months. For the logistic regression analyses, we used the adherence questionnaire as an ordinal scale.

### Psychopathology and global functioning

Information on current psychopathology was obtained with clinician-rated assessment scales, i.e., the Positive and Negative Syndrome Scale (PANSS), Clinician Inventory of Depressive Symptomatology (IDS-C_30_), and Young Mania Rating Scale (YMRS). The PANSS assesses the severity of typical symptoms of schizophrenia [35]; the IDS-C_30_ measures the severity of depressive symptoms [36]; and the YMRS evaluates the severity of mania symptoms [37]. For the cross-diagnostic descriptive analyses, we calculated PANSS, YMRS, and IDS-C_30_ for all participants. For the diagnostic subgroup analyses, we calculated PANSS for the schizophrenia-spectrum disorder group and YMRS and IDS-C_30_ for the bipolar disorder group. Severity of illness was measured with the Clinical Global Impression scale (CGI) [38]. Global Functoning was measured with the Global Assessment of Functioning (GAF) [39].

### Statistical analyses

We calculated cognitive composite scores for three cognitive domains: *learning and memory* (VDS forwards), *executive function* (VDS backwards, TMT B, TMT B-TMT A), and *psychomotor speed* (TMT A, DST). The scores for the cognitive domains were created by generating z-scores of the related variables, positively orienting the partially negative scores and then summing the respective z-scores [40].

Despite the violation of the normal distribution assumption in some variables, the data were analyzed with parametric tests because of the large sample size [41].

The prerequisites of multiple linear regression models were checked: all regression models were tested for the assumptions underlying linear multiple regression and found to adhere to them. A linear relationship was found between the variables, and a check for outliers was performed. Some outliers were found, but because any exclusion of a case from the total sample always involves a loss of power, the outliers were left in the data set. Furthermore, the homoscedasticity of the residuals was confirmed.

As a first step, we performed four multiple linear regression models in the cross-diagnostic sample with the cognitive domains (z-scores) and GAF as the dependent variables and adherence behavior, sex, age, illness duration, number of medications, and diagnosis as the predictors. Because these multiple regression models were significantly driven by the diagnosis, we subsequently performed separate multiple linear regression models for each diagnostic group, with the cognitive domains (z-scores) as the dependent variables and adherence behavior, sex, age, illness duration, number of medications, and symptom scales (PANSS in the schizophrenia-spectrum disorder group and YMRS and IDS-C_30_ in the bipolar disorder group) as the predictors. In model 4 (*psychomotor speed* domain), the heteroscedasticity value was low (Durbin-Watson < 1), indicating unequal variances of the residuals of the variables. Therefore, the HC4 method (heteroscedasticity-consistent standard error estimator) was applied, and robust standard errors were used [42]. The residuals were normally distributed.

In addition, a hierarchical cluster analysis (HCA) was performed to identify subgroups with homogeneous cognitive patterns. We included the z-scores (adjusted for age and sex) of the three cognitive domains *learning and memory*, *executive function*, and *psychomotor speed* as variables. A dendrogram was formed with the Ward’s linkage method and Euclidean distance. The subgroups were determined in an agglomerative manner, and the final number of subgroups (three clusters) was chosen by visually inspecting the dendrogram (see Supplementary Material 2) [43].

After forming the clusters, differences between clusters were analyzed with Chi-square tests and analyses of variance (ANOVA), as appropriate. When significant differences emerged, Levene’s tests were applied to check variance homogeneity, and the groups were evaluated with post hoc comparisons (Bonferroni or Games-Howell, as appropriate).

An alpha value of 0.05 was considered significant. Bonferroni correction for multiple testing was applied for the predictors in the regression models and for Chi-square tests and ANOVA in the cluster comparisons. Corresponding alpha values are indicated in each case. Complete test statistics are displayed in the respective tables. Statistical analyses were performed with IBM SPSS statistics, version 25.0.

## Results

The study sample consisted of 862 participants with a mean [SD] age of 41.9 [12.48] years; 44.8% were female (*n* = 386), and 55.2% male (*n* = 476). Half of the participants (50.9%; *n* = 439) were diagnosed with a schizophrenia-spectrum disorder, and 49.1% (*n* = 423) with bipolar disorder. The descriptive data of the sample are displayed in Table 1.

**Table 1:** Descriptive data of the sample.

	*N*	Minimum	Maximum	Mean	*SD*
Age at first interview	862	18	77	41.99	12.48
Duration of illness, y	815	0	50	12.25	10.08
Medication adherence scale	862	1	6	1.76	1.32
Clinical global impression	859	1	7	4.05	1.01
Global assessment of functioning	862	4	97	57.45	13.45
Number of antidepressants prescribed	862	0	3	0.44	0.61
Number of antipsychotics prescribed	862	0	5	1.38	0.96
Number of mood stabilizers prescribed	862	0	3	0.47	0.60
Number of tranquilizers prescribed	862	0	2	0.21	0.47
Total number of medications prescribed	862	0	8	2.52	1.28
Learning and memory, z-score	862	−3.144	2.851	0.052	0.984
Executive function, z-score	862	−3.258	3.258	0.004	1.004
Psychomotor speed, z-score	862	−2.593	3.275	0.067	0.968
PANSS total sum score	819	30	114	49.45	16.44
IDS-C_30_ sum score	774	0	55	12.99	10.52
YMRS sum score	841	0	36	3.12	4.97

*IDS-C*_*30*_ Inventory of Depressive Symptomatology, clinician-rated, *PANSS* Positive and Negative Syndrome Scale, *SD* standard deviation, *YMRS* Young Mania Rating Scale.

### Cross-diagnostic analyses

All four multiple linear regression models showed a significant effect with the predictors adherence behavior, sex, age, duration of illness, number of medications, and diagnosis: model 1, GAF (*F*(5, 808) = 25.51, *p* < 0.001, *R*^*2*^_*a*_ = 0.159); model 2, cognitive domain *learning and memory* (*F*(5, 808) = 4.28, *p* < 0.001, *R*^*2*^_*a*_ = 0.02); model 3, cognitive domain *executive function* (*F*(5, 808) = 16.70, *p* < 0.001, *R*^*2*^_*a*_ = 0.10); and model 4, cognitive domain *psychomotor speed* (*F*(5, 808) = 34.05, *p* < 0.001, *R*^*2*^_*a*_ = 0.196). After correcting for multiple testing (all Bonferroni adjustments, 0.05/6 = *p* < 0.008), the results indicated the following (see Table 2): The association between the predictors and GAF was significantly driven by adherence behavior, number of prescribed medications, and diagnosis, whereby higher adherence behavior (*p* < 0.001), less prescribed medication (*p* < 0.001), and a diagnosis of bipolar disorder (*p* < 0.001) predicted better global functioning; the association between the predictors and *learning and memory* was significantly driven by age and diagnosis, whereby younger age (*p* = 0.002) and a diagnosis of bipolar disorder (*p* < 0.001) predicted better performance; and the association between the predictors *executive function* was significantly driven by age, number of prescribed medications, and diagnosis, whereby younger age (*p* < 0.001), less prescribed medication (*p* = 0.002), and a diagnosis of bipolar disorder (*p* < 0.001) predicted better performance; and the association between the predictors and *psychomotor speed* was significantly driven by sex, age, number of prescribed medications, and diagnosis, whereby female sex (*p* = 0.004), younger age (*p* < 0.001), less prescribed medication (*p* < 0.001), and a diagnosis of bipolar disorder (*p* < 0.001) predicted better performance.

**Table 2:** Multiple linear regression analyses in the whole sample of patients with schizophrenia-spectrum and bipolar disorders, with cognitive performance as the dependent variable and adherence behavior, sex, age, duration of illness, number of prescribed medications, and diagnosis as the predictors.

Patients with schizophrenia-spectrum and bipolar disorders										
		Unstandardized		Standardized	*t* value	*p* value	R^2^	R^2^_a_	df1	df2
		USC B	SE	SC B						
Learning and memory						<0.001*	0.031	0.024	5	808
	Adherence scale	−0.03	0.03	−0.04	−1.10	0.270				
	Sex	−0.02	0.07	−0.01	−0.30	0.765				
	Age	−0.01	0.00	−0.13	−3.09	0.002**				
	Illness duration	0.00	0.00	0.01	0.13	0.899				
	No. of medications	−0.04	0.03	−0.05	−1.52	0.130				
	Diagnosis	0.27	0.07	0.14	3.78	<0.001*				
Executive function						<0.001*	0.110	0.104	5	808
	Adherence scale	0.00	0.026	−0.001	−0.028	0.978				
	Sex	−0.01	0.067	−0.004	−0.121	0.904				
	Age	−0.02	0.003	−0.302	−7.35	<0.001**				
	Illness duration	0.00	0.004	−0.001	−0.037	0.971				
	No. of medications	−0.08	0.026	−0.103	−3.042	0.002**				
	Diagnosis	0.34	0.068	0.171	4.946	<0.001**				
Psychomotor speed						<0.001*	0.202	0.196	5	808
	Adherence scale	−0.038	0.023^a^		−1.59	0.111				
	Sex	0.173	0.061^a^		2.80	0.005**				
	Age	−0.028	0.003^a^		−9.88	<0.001**				
	Illness duration	−0.004	0.004^a^		−1.20	0.23				
	No. of medications	−0.092	0.024^a^		−3.88	<0.001**				
	Diagnosis	0.493	0.062^a^		7.75	<0.001**				
GAF						<0.001*	0.159	0.153	5	808
	Adherence scale	−1.424	0.335	−0.141	−4.253	<0.001**				
	Sex	0.885	0.873	0.033	1.014	0.311				
	Age	0.046	0.043	0.043	1.072	0.284				
	Illness duration	−0.028	0.052	−0.021	−0.546	0.585				
	No. of medications	−2.226	0.343	−0.213	−6.493	<0.001**				
	Diagnosis	7.693	0.893	0.29	8.618	<0.001**				

*df* degrees of freedom, *HC* heteroscedasticity consistent, *SC B* standardized coefficient, *SE* standard error, *USC B* unstandardized coefficient β.

*p < 0.05 significant; **p < 0.008 significant (Bonferroni corrected for multiple testing).

^a^Robust standard errors after HC4 correction.

### Separate analyses in the diagnostic groups

In the schizophrenia-spectrum disorder group, the regression model showed a statistically significant effect of the predictors adherence behavior, sex, age, duration of illness, number of medications, and PANSS (sum score of positive symptoms, sum score of negative symptoms, and sum score of general symptoms) on the cognitive domains *learning and memory* (*F*(7, 404) = 4.71, *p* < 0.001, *R*^*2*^_*a*_ = 0.067), *executive function* (*F*(7, 404) = 10.93, *p* < 0.001, *R*^*2*^_*a*_ = 0.162), and *psychomotor speed* (*F*(7, 404) = 12.79, *p* < 0.001, *R*^*2*^_*a*_ = 0.186). After correcting for multiple testing (all Bonferroni adjustments 0.05/8 = *p* < 0.006), the results indicated that the association between the predictors and *learning and memory* was significantly driven by the PANSS negative symptoms sum score, i.e., a lower negative symptoms score (*p* < 0.001) predicted better performance in this domain; that the association between the predictors and *executive function* was significantly driven by age and the PANSS negative symptoms sum score, i.e., lower age (*p* < 0.001) and fewer negative symptoms (*p* < 0.001) predicted better performance in this domain; and that the association between the predictors and *psychomotor speed* was significantly driven by age and the PANSS general psychopathology sum score, i.e., lower age (*p* < 0.001) and fewer negative symptoms (*p* < 0.001) predicted better performance in this domain (Table 3).

**Table 3:** Multiple linear regression analyses in the group of patients with schizophrenia-spectrum disorder with cognitive performance as the dependent variable and adherence behavior, sex, age, duration of illness, number of medications, and Positive and Negative Syndrome Scale scores as the predictors.

Group of patients with schizophrenia-spectrum disorder										
		Unstandardized		Standardized	*t* value	*p* value	R^2^	R^2^_a_	df1	df2
		USC B	SE	SC B						
Learning and memory						<0.001*	0.085	0.067	7	404
	Adherence scale	−0.02	0.04	−0.03	−0.48	0.630				
	Sex	−0.02	0.10	−0.01	−0.16	0.875				
	Age	−0.02	0.01	−0.17	−2.73	0.007				
	Illness duration	0.00	0.01	−0.04	−0.67	0.501				
	No. of medications	0.00	0.04	−0.01	−0.11	0.910				
	PANSS positive sum score	−0.02	0.01	−0.13	−1.89	0.059				
	PANSS negative sum score	−0.04	0.01	−0.23	−3.57	<0.001**				
	PANSS general sum score	0.01	0.01	0.11	1.28	0.202				
Executive function						<0.001*	0.178	0.162	7	404
	Adherence scale	0.01	0.03	0.02	0.35	0.724				
	Sex	−0.03	0.09	−0.01	−0.29	0.772				
	Age	−0.02	0.01	−0.25	−4.12	<0.001**				
	Illness duration	−0.01	0.01	−0.13	−2.13	0.034				
	No. of medications	0.00	0.04	0.00	0.05	0.961				
	PANSS positive sum score	−0.02	0.01	−0.12	−1.84	0.066				
	PANSS negative sum score	−0.04	0.01	−0.26	−4.17	<0.001**				
	PANSS general sum score	0.01	0.01	0.05	0.62	0.534				
Psychomotor speed						<0.001*	0.202	0.186	7	404
	Adherence scale	0.01	0.03^a^		0.47	0.636				
	Sex	0.16	0.08^a^		1.92	0.055				
	Age	−0.02	0.00^a^		−4.64	<0.001**				
	Illness duration	−0.01	0.01^a^		−2.54	0.012				
	No. of medications	0.00	0.03^a^		0.10	0.921				
	PANSS positive sum score	−0.01	0.01^a^		−1.06	0.289				
	PANSS negative sum score	−0.04	0.01^a^		−4.60	<0.001**				
	PANSS general sum score	0.00	0.01^a^		−0.11	0.914				

*df* degrees of freedom, *HC* heteroscedasticity consistent, *PANSS* Positive and Negative Symptom Scale, *SC B* standardized coefficient, *SE* standard error, *USC B* unstandardized coefficient β.

*p < 0.05 significant; **p < 0.008 significant (Bonferroni corrected for multiple testing).

^a^Robust standard errors after HC4 correction.

In the bipolar disorder group, the regression model showed a statistically significant effect of the predictors adherence behavior, sex, age, duration of illness, number of medications, depressive symptoms (IDS-C_30_ sum score), and manic symptoms (YMRS sum score) on the cognitive domains *executive function* (*F*(6, 328) = 8.09, *p* < 0.001, *R*^*2*^_*a*_ = 0.129) and *psychomotor speed* (*F*(6, 328) = 15.88, *p* < 0.001, *R*^*2*^_*a*_ = 0.237). After correcting for multiple testing (all Bonferroni adjustments 0.05/8 = *p* < 0.007), the results indicated that the association between the predictors and *executive function* was significantly driven by age, number of prescribed medications, and manic symptoms, i.e., lower age (*p* < 0.001), less prescribed medication (*p* = 0.005), and fewer manic symptoms (*p* < 0.001) predicted better performance in this domain, and that the association between the predictors and *psychomotor speed* was significantly driven by age and number of prescribed medications, i.e., lower age (*p* < 0.001) and fewer prescribed medications (*p* = 0.001) predicted better performance in this domain (Table 4). No significant results were obtained in the regression model that examined the influence of the predictors adherence behavior, sex, age, duration of illness, number of medications, depressive symptoms (IDS-C_30_ sum score), and manic symptoms (YMRS sum score) on the cognitive domain *learning and memory* (*F*(6, 328) = 1.12, *p* = 0.265, *R*^*2*^_*a*_ = 0.02; Table 4).

**Table 4:** Multiple linear regression analyses in the group of patients with bipolar disorder with cognitive performance as the dependent variable and adherence behavior, sex, age, duration of illness, number of medications, Young Mania Rating Scale, and Clinician Inventory of Depressive Symptomatology (IDS-C_30_) as the predictors.

Group of patients with bipolar disorder										
		Unstandardized		Standardized	*t* value	*p* value	R^2^	R^2^_a_	df1	df2
		USC B	SE	SC B						
Learning and memory						0.265	0.023	0.002	6	328
	Adherence scale	−0.02	0.05	−0.03	−0.47	0.639				
	Sex	−0.10	0.11	−0.05	−0.94	0.350				
	Age	−0.01	0.01	−0.11	−1.68	0.093				
	Illness duration	0.00	0.01	0.02	0.27	0.786				
	No. of medications	−0.05	0.04	−0.07	−1.25	0.211				
	YMRS sum score	−0.01	0.01	−0.06	−1.10	0.274				
	IDS-C_30_ sum score	0.00	0.01	−0.04	−0.66	0.509				
Executive function						<0.001*	0.147	0.129	6	328
	Adherence scale	0.04	0.05	0.05	0.86	0.393				
	Sex	−0.03	0.11	−0.02	−0.29	0.770				
	Age	−0.03	0.01	−0.33	−5.58	<0.001**				
	Illness duration	0.01	0.01	0.08	1.32	0.188				
	No. of medications	−0.12	0.04	−0.15	−2.85	0.005**				
	YMRS sum score	−0.03	0.01	−0.19	−3.62	<0.001**				
	IDS-C_30_ sum score	0.00	0.01	−0.05	−0.88	0.377				
Psychomotor speed						<0.001*	0.253	0.237	6	328
	Adherence scale	−0.03	0.05^a^		−0.53	0.600				
	Sex	0.09	0.10^a^		0.98	0.329				
	Age	−0.03	0.00^a^		−8.22	<0.001**				
	Illness duration	0.00	0.01^a^		−0.58	0.564				
	No. of medications	−0.12	0.04^a^		−3.08	0.002**				
	YMRS sum score	−0.01	0.01^a^		−1.51	0.131				
	IDS-C_30_ sum score	−0.01	0.01^a^		−2.25	0.025				

*df* degrees of freedom, *HC* heteroscedasticity consistent, *IDS-C*_*30*_ Inventory of Depressive Symptomatology, clinician rated, *SC B* standardized coefficient, *SE* standard error, *USC B* unstandardized coefficient β, *YMRS* Young Mania Rating Scale.

*p < 0.05 significant; **p < 0.008 significant (Bonferroni corrected for multiple testing).

^a^Robust standard errors after HC4 correction.

### Cluster analysis

**Fig. 1:**
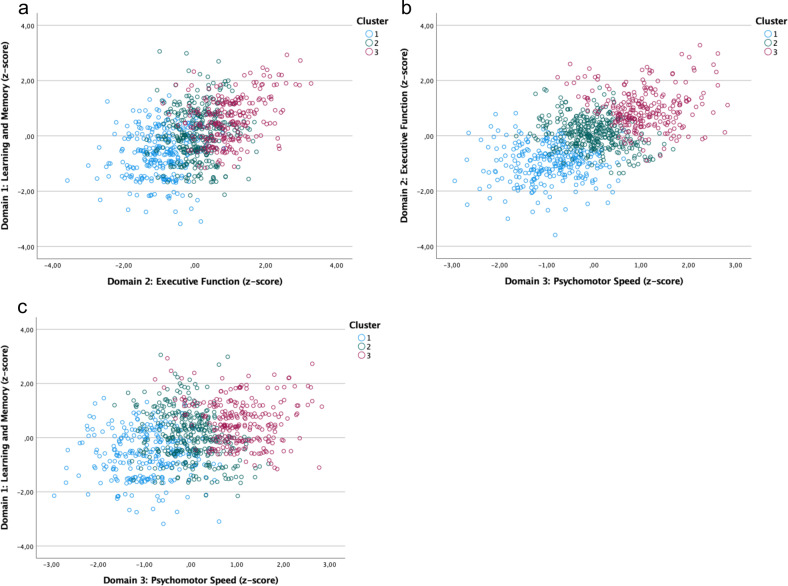
Cross-diagnostic clusters of cognitive performance. Scatter plots showing the distribution, with color-coded cluster designations, of the following cognitive domains: **a** learning and memory and executive function, **b** executive function and psychomotor speed, and **c** learning and memory and psychomotor speed.

The HCA resulted in three clusters: Cluster 1 comprised 32.4% (*n* = 279) of the patients; cluster 2, 40.0% (*n* = 336); and cluster 3, 28.6% (*n* = 247; Fig. 1). The ANOVA and the post hoc testing of the neuropsychological raw data showed significant differences between the three clusters in the following measures (after Bonferroni correction for multiple testing; *p* < 0.007): TMT A (*F*(2, 859) = 211.38, *p* < 0.001), TMT B (*F*(2, 859) = 353.05, *p* < 0.001), TMT B errors (*F*(2, 851) = 37.43, *p* < 0.001), VDS forwards (*F*(2, 859) = 131.76, *p* < 0.001), VDS backwards (*F*(2, 859) = 213.91, *p* < 0.001), and DST (*F*(2, 859) = 394.87, *p* < 0.001). Differences in TMT A errors (*F*(2, 849) = 4.75, *p* = 0.009) was not significant. Cluster 1 had the poorest cognitive performance, and cluster 3 the best.

Furthermore, significant differences were found between the clusters regarding diagnosis, premorbid IQ, number of medication, GAF, CGI, and PANSS. While no significant differences were found between the clusters regarding, illness duration, adherence behavior and IDS-C_30_, and YMRS sum scores (Table 5).

**Table 5:** Descriptive analyses of the three clusters.

	Cluster 1 (*n* = 279)	Cluster 2 (*n* = 336)	Cluster 3 (*n* = 247)	Statistics	*p* value	Post hoc analysis
	Mean (S*D*) or *n* (%)	Mean (*SD*) or *n* (%)	Mean (*SD*) or *n* (%)			
Illness duration, y	13.37 (10.95)	11.75 (9.78)	11.61 (9.30)	*F*(2, 812) = 2.54	<0.08	
Diagnosis				X^2^(2) = 48.53	<0.001*	
Schizophrenia-spectrum disorder	184 (42.0)	167 (38.0)	88 (20.0)			1 > 3
Bipolar disorder	95 (22.5)	169 (39.9)	159 (37.6)			1 < 3
Premorbid IQ	25.70 (5.44)	28.30 (4.33)	30.20 (3.92)	*F*(2, 756) = 55.64	<0.001*	1 < 2, 1 < 3, 2 < 3
Adherence Scale	1.78 (1.42)	1.82 (1.37)	1.68 (1.15)	*F*(2, 859) = 0.81	0.444	
No. of medications	2.70 (1.36)	2.54 (1.34)	2.30 (1.08)	*F*(2, 859) = 6.22	0.002*	1 > 3
GAF score	52.62 (12.54)	58.60 (13.45)	61.35 (13.16)	*F*(2, 859) = 31.73	<0.001*	1 < 2, 1 < 3
CGI score	4.41 (0.90)	3.99 (1.03)	3.73 (0.99)	*F*(2, 856) = 32.73	<0.001*	1 > 2, 1 > 3
PANSS						
PANSS positive sum score	13.10 (5.50)	10.85 (4.46)	9.61 (3.87)	*F*(2, 853) = 38.05	<0.001*	1 > 2, 1 > 3, 2 > 3
PANSS negative sum score	14.73 (6.46)	12.04 (5.71)	10.65 (4.21)	*F*(2, 844) = 36.23	<0.001*	1 > 2, 1 > 3, 2 > 3
PANSS general sum score	28.55 (8.57)	25.10 (7.73)	23.15 (6.61)	*F*(2, 831) = 32.30	<0.001*	1 > 2, 1 > 3
PANSS total sum score	56.66 (17.77)	48.03 (15.60)	43.34 (12.66)	*F*(2, 816) = 47.38	<0.001*	1 > 2, 1 > 3, 2 > 3
IDS-C_30_ sum score	14.01 (10.85)	12.93 (10.54)	11.93 (10.05)	*F*(2, 771) = 2.34	0.097	
YMRS sum score	3.61 (5.22)	3.01 (4.94)	2.72 (4.70)	*F*(2, 838) = 2.18	0.113	

z-scores were adjusted for age and sex for cluster analysis.

*CGI* Clinical Global Impression, *GAF* Global Assessment of Functioning, *IDS-C*_*30*_ Inventory of Depressive Symptomatology, clinician rated, *PANSS* Positive and Negative Syndrome Scale, *SD* Standard deviation, *y* years, *YMRS* Young Mania Rating Scale.

*p < 0.003 significant (Bonferroni corrected for multiple testing).

## Discussion

Evidence for an association between medication adherence and cognitive impairment remains sparse, and results of previous studies are conflicting [28]. Therefore, we addressed this research question in a large, well-phenotyped, cross-diagnostic sample of patients from the PsyCourse Study. We aimed to determine whether medication (non)adherence influences cognitive performance in patients with schizophrenia-spectrum and bipolar disorders and whether a specific, disease-related cognitive pattern affects medication adherence.

We found positive effects of medication adherence on global functioning. However, conclusions about the direction of influence cannot be drawn from our study. Much research shows that good medication adherence eases the symptom load in psychiatric disorders, contributes to a positive clinical outcome, and leads to better global functioning and perceived quality of life [44–46]. Yet, we found no direct positive effects of medication adherence on cognitive function. Cognitive performance seemed to be influenced by other well-known factors. According to previous research, we found that higher age may be the most prominent factor associated with cognitive deterioration. Extensive research has shown that the older we get, the more structural brain damage occurs and the poorer our cognitive performance becomes [47]. In line with recent studies, we showed that a diagnosis of schizophrenia-spectrum or bipolar disorder has an impact on cognitive performance and that individuals with bipolar disorder have better cognitive functioning than individuals with schizophrenia-spectrum disorders [19].

To further investigate this finding, we repeated the analysis separately in the diagnostic groups and added factors relevant to symptomatic burden. In participants with a schizophrenia-spectrum disorder, performance in *learning and memory* and *executive function* was significantly driven by the PANSS negative symptom score, i.e., the more negative symptoms participants had, the worse their performance was. This finding is consistent with previous studies, which showed that the negative symptom dimension appears to have the strongest relationship with cognitive performance [5] and both negative symptoms and cognitive deficits are hard to address with the available medication. A recent meta-analysis showed heterogeneous effects of the different antipsychotics on cognitive domains; when compared to other antipsychotics, substances like amisulpride or quetiapine performed better on cognitive scores, yet other antipsychotics like haloperidol and clozapine lead to poorer performance [48]. In our study, we did not focus on the differential effects of the different antipsychotics. We can only make a statement about the lacking association of medication adherence and cognitive function, but cannot draw a conclusion of the specific pharmaceutical effects on cognition in general. Non-pharmaceutical strategies like cognitive remediation therapy (CRT) might be a promising approach to enhance cognitive performance [49]; however, evidence that CRT might also improve medication adherence is sparse and needs further investigation [50].

Results in participants with bipolar disorder were different. The variance in the domain *learning and memory* could not be explained by the factors of our regression model, so deficits in this domain may depend on other factors that we did not consider in our model, such as hippocampal dysfunction and genetic or structural abnormalities [51–53]. *Executive function* was rather associated with age, the sum of prescribed medication, and a higher load of manic symptoms. Mania includes difficulties in emotionality, emotion regulation, and emotion-relevant impulsivity [54], and response inhibition is often reduced during phases of mania, which can impair executive functioning. Therefore, our results support the existing hypothesis that some cognitive deficits in bipolar disorder depend rather on state than trait [17]. The domain *psychomotor speed* was associated with age and the amount of medication. Mood stabilizers, antipsychotics and anticonvulsants often have anticholinergic side effects and can impair cognitive performance [55]. Lithium is sometimes described as a neuroprotective drug, yet showed a moderate negative effect on cognition in meta-analyses [56, 57]. Although the total number of prescribed medications appears to play an important role in cognitive performance, we found no evidence that medication adherence itself affected cognitive performance in patients with bipolar disorder. Unfortunately, our study is lacking more detailed information on the prescribed medication and is therefore also limited in the differentiated interpretation of the results. But overall, this finding is consistent with the knowledge that current drugs for bipolar disorder are not capable of rescuing cognitive performance [58].

Beyond the diagnostic groups, we wanted to investigate whether specific cross-diagnostic cognitive clusters exist and whether patients in different clusters differ in their adherence behavior. In our explorative cluster analysis, we identified three cognitive clusters with severe (cluster 1), moderate (cluster 2), and mild (cluster 3) cognitive impairment. Cluster 1 comprised mostly patients with schizophrenia-spectrum disorders, and cluster 3, mostly patients with bipolar disorder. In the moderately impaired group (cluster 2), neither of the diagnoses was more prevalent than the other. Previous studies showed that schizophrenia and bipolar disorder may share a common genetic cause and resemble each other in symptomology [4, 59], so cluster 2 might represent the overlapping endophenotype of schizophrenia and bipolar disorder. In general, those with severe cognitive impairment (cluster 1) were more impaired overall, experienced more psychotic symptoms and had a lower general IQ. In line with other clustering approaches, they differed significantly with regard to global function and disease severity [60]. More importantly, we investigated whether the patients in the three cognitive clusters differed in their adherence behavior and found no differences. Whether or not a patient takes his or her medication, appears to depend on factors other than cognitive performance, and vice versa; a better or worse medication adherence does not appear to impact cognitive performance.

Despite the new findings and strengths of our study (i.e., large sample size and well-phenotyped cross-diagnostic sample), some limitations must be addressed. We investigated medication adherence only with a non-standardized self-report questionnaire that focused on the regularity of medication intake. The questionnaire is self-constructed and has not been pre-tested and validated. This obstructs the replication of the study and impacts its comparison to other studies. Furthermore, we evaluated adherence behavior and the other variables simultaneously, so the variables may have interacted with each other and cannot be interpreted independently. We included the total number of currently prescribed medications as a predictor in the model. The collection of more detailed information on the pharmacological treatment (such as molecule, generation of antipsychotics, chlorpromazine equivalents, and plasma levels) would have helped to interpret these somewhat counterintuitive results in a more informed way. Especially the route of administration (injectable vs oral) might have a major impact on medication adherence and therefore mediated the results [61]. These information should be of interest in future investigations.

In conclusion, we were able to answer our research questions, as follows: 1) medication adherence does not influence cognitive but does affect global functioning in patients with schizophrenia-spectrum and bipolar disorders; (2) in both diagnostic groups, cognitive function is not influenced by medication adherence but is influenced by other factors; and (3) cognitive subgroups exist independent of diagnoses, but the patients in these clusters do not differ in their adherence behavior. Taken together, these findings indicate that clinicians’ hopes that good medication adherence itself can help improve cognitive performance cannot be generalized. However, non-pharmaceutical therapeutic approaches have been proven to have positive effects on cognitive performance, such as behavioral therapy, cognitive remediation therapy and neuropsychological cognitive training [62, 63], and our study underlines the importance of further investigating the long-term effects of these non-drug strategies on cognitive function in large-scale studies.

## Supplementary information

Supplementary Material 1.

Supplementary Material 2.
