# Supplementary material for: Medication adherence and cognitive performance in schizophrenia-spectrum and bipolar disorder: results from the PsyCourse Study
Source: Transl Psychiatry. 2023 Mar 25;13:99. doi: 10.1038/s41398-023-02373-x (PMC10039892; doi:10.1038/s41398-023-02373-x)
Supplement: Supplementary file 1 — Supplementary Material 1. [file 41398_2023_2373_MOESM1_ESM.docx]

**Supplementary Material 1.** Description of the neurocognitive test instruments.

Global Assessment of Functioning

The Global Assessment of Functioning (GAF), measures psychological, social, and occupational functioning on a continuous scale ranging from one to 100. A score of one to 10 indicates persistent and severe impairment, and a score of 91 to 100, no impairment ^1^. According to Endicott et al., most psychiatric outpatients have a score of 31 to 70 and most inpatients, a score of one to 40 ^2^.

Trail Making Test

The Trail Making Test (TMT) measures multiple cognitive domains, such as visual attention and psychomotor speed (parts A and B) and task switching (part B), and therefore provides a good reflection of executive function. Participants are required to connect digits (part A) or alternately connect digits and letters (part B) in increasing order as quickly and accurately as possible. The time taken to complete each part of the test is measured. An estimate of the task-switching process (a component of executive function) is obtained by subtracting the time for part A from that for part B ^3^.

Verbal Digit Span

The Verbal Digit Span (VDS) measures short-term (forward digit span) and working memory (backward digit span), as a part of executive function. The participant is asked to repeat digits verbally presented by the interviewer, first forwards, then backwards. One point is given for each correctly recalled string of digits, and the interviewer continues until the participant makes mistakes in recalling strings of the same length. The final score is built by adding all the points ^4^.

Digit Symbol Test

The Digit Symbol Test (DST) is a well-known test for measuring psychomotor speed. Participants receive a test sheet that has rows of numbers, an empty space below each number, and a corresponding number-symbol key at the top. They are required to use the key to fill in as many symbols as possible below the numbers during a 120-second period. The number of correct symbols is the final score ^5^.

Verbal intelligence

The multiple-choice vocabulary intelligence test (MWT-B) was used to assess verbal intelligence, an approximate measure of general intelligence ^6^. This test contains 37 sets of five words each, four of which are “artificial words,” i.e., they do not exist in German. Participants are instructed to mark the correct word, and the number of correctly recognized words forms the final score.

**References**

1 Aas IM. Guidelines for rating Global Assessment of Functioning (GAF). *Ann Gen Psychiatry* 2011; **10**: 2.

2 Endicott J. The Global Assessment Scale: A Procedure for Measuring Overall Severity of Psychiatric Disturbance. *Arch Gen Psychiatry* 1976; **33**: 766.

3 Tischler L, Petermann F. Trail Making Test (TMT). *Zeitschrift für Psychiatrie, Psychologie und Psychotherapie* 2010; **58**: 79–81.

4 Wahlstrom D, Weiss LG, Saklofske DH. Practical Issues in WISC-V Administration and Scoring. In: *WISC-V Assessment and Interpretation*. Elsevier, 2016, pp 25–62.

5 Molz C, Schulze R, Schroeders U, Wilhelm O. TBS-TK Rezensionen: Wechsler Intelligenztest für Erwachsene WIE. Deutschsprachige Bearbeitung und Adaptation des WAIS-III von David Wechsler. *Psychologische Rundschau* 2010; **61**: 229–230.

6 Lehrl S. *Mehrfachwahl-Wortschatz-Intelligenztest: MWT-B*. 5., unveränd. Aufl. Spitta: Balingen, 2005.
