# Supplementary figures and images for: Medication adherence and cognitive performance in schizophrenia-spectrum and bipolar disorder: results from the PsyCourse Study
Source: Transl Psychiatry. 2023 Mar 25;13:99. doi: 10.1038/s41398-023-02373-x (PMC10039892; doi:10.1038/s41398-023-02373-x)

**Supplementary Material 2.** Dendrogram using ward-linkage

**
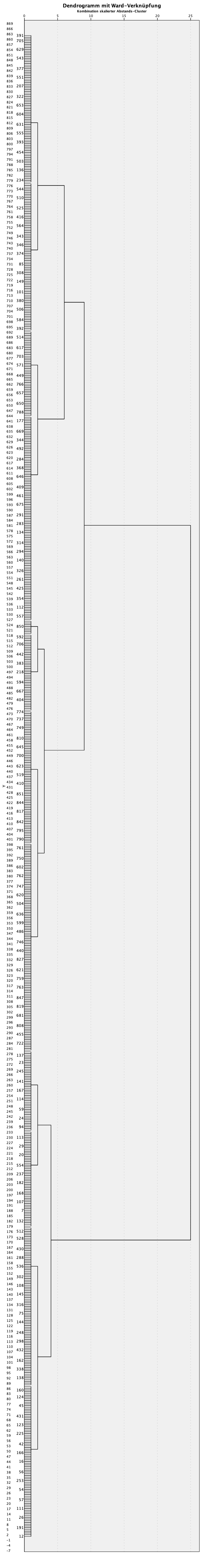
**

Supplement: Supplementary file 2 — Supplementary Material 2. [file 41398_2023_2373_MOESM2_ESM.docx]
